# Promoter Hypermethylation of *LATS2* Gene in Oral Squamous Cell Carcinoma (OSCC) among North Indian Population

**DOI:** 10.31557/APJCP.2020.21.5.1283

**Published:** 2020-05

**Authors:** Harsh Goel, Saloni Singhal, Runjhun Mathur, Saima Syeda, Rishi Kumar Gupta, Anshuman Kumar, Anju Shrivastava, Abhimanyu Kumar Jha

**Affiliations:** 1 *Department of Biotechnology, Institute of Applied Medicines and Research Ghaziabad, Uttar Pradesh, India. *; 2 *Dr. A.P.J. Abdul Kalam Technical University, Lucknow, Uttar Pradesh, India. *; 3 *Department of Zoology, Delhi University, India. *; 4 *Sh. Jagannath Charitable Cancer Hospital, Ghaziabad, Uttar Pradesh, India. *; 5 *Dharamshila Cancer Hospital and Research Centre, New Delhi, India. *

**Keywords:** Oral squamous cell carcinoma (OSCC), epigenetic changes, LATS2 gene, promoter hypermethylation

## Abstract

Large Tumor Suppressor (*LATS2*) gene are Tumor Suppressor gene, linked with epigenetic modifications. LATS2 promoter hypermethylation is an important epigenetic silencing mechanism leading to cancer. Cancer is the most common, vicious and dangerously increasing diseases of the world today, associated with high morbidity and mortality. Oral cancers (OC) are the blazing universal dilemma and is the sixth most frequent cancer observed in Indian population. Tobacco consumption is the main cause of the increase in OSCC. The association between *LATS2* in the pathogenesis of cancers propose that their combination might be studied as a possible molecular marker for particular subgroups of patients. Therefore, the present study tried to investigate whether *LATS2* promoter methylation was associated with oral squamous cell carcinoma (OSCC) in North Indian subjects. DNA methylation quantitative studies of *LATS2* Tumor Suppressor genes were performed by methylation-specific polymerase chain reaction (MSP). 38 out of 70 patients (55 %) were found to be methylated for *LATS2* gene, a statistically significant result was obtained (p-value < 0.005) for *LATS2* genes. The results suggest that epigenetic changes may be related to the down-regulation of *LATS2* expression. It can be concluded that *LATS2* gene plays a significant role in the diagnosis of cancer and provide a better alternative as a diagnostic biomarker. Our data infer that a low *LATS2* expression due to methylation may contribute to the cancer progression and could be useful for the diagnosis of OSCC. Therefore, investigation of promoter methylation in such genes may provide a biomarker which may prove to be useful in early detection of Oral Cancer.

## Introduction

In developing countries, Oral Squamous Cell Carcinoma (OSCC) is the most popular epithelial malignancy influencing the oral cavity (Parkin et al., 2001). OSCC accounts for more than 90 % of all oral neoplasms (Choi and Myers 2008). The prevalence rate of oral cancer is 2.7 and 1.5 per 100,000 in men and women, respectively (Kordi-Tamandani et al., 2010). Growth of OSCC is a multistep process, resulting from a combination of genetic susceptibility and environmental risk factors including tobacco and alcohol consumption, chronic inflammation and viral infection (Chien et al., 2013). Oncogenes and Tumor Suppressor genes promote Tumorigenesis (Hanahan and Weinberg 2000). The down-regulation of Tumor Suppressor genes is introduced through several epigenetic modifications, mutations, loss of heterozygosity and deletions (Perez-Sayans et al., 2009). The epigenetic changes refer to any mitotically heritable alteration in gene expression without changes of the DNA sequence. These changes occur more commonly than gene mutation. 

Epigenetic changes include DNA methylation, histone modifications, and RNA-mediated silencing. Disturbance of any of these three different mutually strengthening epigenetic mechanisms leads to dysregulation of gene expression, resulting in cancer progression and other epigenetic diseases The epigenetic suppression of genes takes place through methylation of CpG islands or histone modifications such as methylation of histone 3, lysine 27 (H3K27) (Rad et al., 2016). Large Tumor Suppressor gene 2 (*LATS2*), which located on the 6q25.1 and 13q12.11 chromosomes, are important Tumor Suppressor genes in the cell cycle control and DDR signaling (Najafi et al., 2016). *LATS2* localizes to the mitotic apparatus and regulate cell cycle through G2-M arrest and G1-S arrest, respectively (Xia et al., 2002; Li et al., 2003). *LATS2* genes are the main Tumor Suppressors of the Hippo signaling pathway which has extensive effects on normal cell fate and Tumorigenesis (Aqeilan 2013). Furthermore, in response to DNA damage, *LATS2* serves as a Tumor Suppressor in Chk1-*LATS2*–14–3-3 and Chk1– *LATS2*–p21 axes (Scrace and O’Neill 2012; Okada et al., 2011; Suzuki et al., 2013). Deregulation of *LATS2* genes through methylation have been crucial in several malignancies such as lung cancer, breast cancer, astrocytoma and colorectal cancer (Sasaki et al., 2010; Jiang et al., 2006; Takahashi et al., 2005; Wierzbicki et al., 2013). The aim of the present study was to investigate the status of *LATS2* promoter methylation in OSCC patients.

## Materials and Methods

*Sample collection*

Blood and serum samples (70) were collected with the informed permission of patients diagnosed with OSCC after taking the necessary Ethical clearance from Dharamshila Cancer Hospital and Research Centre, Delhi, India. The blood samples (20) from healthy individuals (as controls) were also obtained. The samples were further used for DNA extraction.

*DNA Extraction*

Cells obtained from tissue biopsies and blood samples were lysed in digestion buffer (10 mM Tris-HCl, pH 8.0, 10 mM EDTA, 150 mM NaCl and 2% SDS) containing proteinase K (0.2 mg/ml). DNA was then purified using the standard phenol-chloroform extraction and ethanol precipitation.

*Sodium Bisulfite Modification*

This method allows the definite study of methylation in a certain region by converting all non-methylated cytosine into uracil, while methylated cytosines remain unchanged. DNA isolated from blood samples was modified with sodium bisulfite using the agarose bead method.

*Methylation-Specific PCR (MS-PCR)*

The amplification of the bisulfite-treated DNA was done through methylation-specific PCR, MS-PCR was carried out using specific primers for methylation and unmethylation for the *LATS2* genes (Table 1). MSP was performed to check the promoter hypermethylation of *LATS2* gene in OSCC patients among north Indian population (Figure 2 and Figure 3). Photomicrograph representing 2.5% agarose gel electrophoresis of PCR amplified *LATS2* gene that was carried out in 1X TAE buffer at a voltage supply of 80 V. 

Following steps and temperature conditions were used in the process: the initial denaturation was carried out at 95°C for 10 mins. The PCR cycles consisting of denaturation at 95°C for 40 sec, annealing at 65°C for 40 sec and elongation step was done at 72°C for 40 sec for 35 cycles. The final extension was carried out at 72°C for 10 min.

*Statistical Analysis*

The hypermethylation of the promoter region of *LATS2* gene of OSCC patients was statistically analyzed through stat calc of Epiinfo tool version 7.2 which was used for computing Odds ratio (OR) and 95% confidence interval (CI) through Chi-square test.

**Figure 1:**
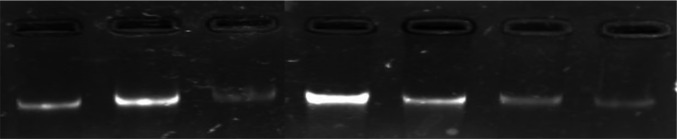
Qualitative Analysis of DNA Isolated from Blood and Serum Samples of OSCC Patients and Controls

• Lane No 1-4: DNA of OSCC patients

• Lane No 5-7: DNA of healthy individuals (controls).

**Table 1:** Sequence of the Primers for *LATS2 *Gene

Name of Gene	Sequences (5′–3′)	Annealing tem (°C)	Product size
*LATS2 M*	F: ATTTCGGTTTATTGTAATTTTC	55 °C	148 bp
	R: AACCAACATAATAAAACCCCG		
*LATS2 U*	F: TTTGTTTTTT GGGTTTAAGT	55 °C	130 bp
	R: CCAACATAATA AAACCCCA		

M, Methylated; U, Unmethylated

**Figure 2:**
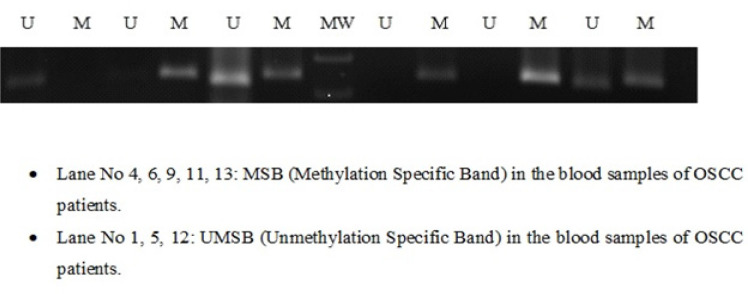
MSB (Methylation Specific Band) and UMSB (Unmethylation Specific Band) of LATS2 in Blood Samples of OSCC Patients

**Table 2:** Frequency of Methylation and Unmethylation of *LATS2* Gene with the Relative Risk of OSCC in Patients and Healthy Controls

Gene Methylation status	Methylated (%)	Unmethylated (%)	OR	95%CI	*P* value
*LATS2* in Patient	38 (55%)	32 (45%)	1.83	1.29- 2.59	0.0007
Samples (N=70)					
*LATS2* in Control (N=20)	0 (0.0%)	20 (100%)	0	0	0.0001

**Table 3:** Frequency Table of Methylation of *LATS2* and Tobacco Chewing Status in OSCC Patients and Healthy Controls

Gene methylation status	Methylated (%)	OR (95% CI)	*P*-value
*LATS2* in Patients Samples (N= 60)	40 (66.6)	1.40 (1.005-1.949)	0.01
*LATS2* in Control Samples (N= 8)	0	0	0.0001

**Figure 3:**
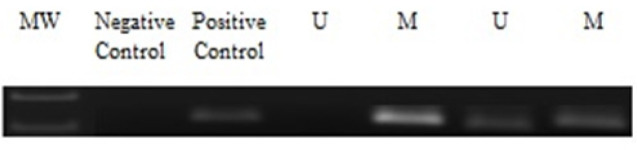
Gel Image with Positive and Negative Control

• Lane No 1: Molecular Marker

• Lane No 2: Negative Control (H2O)

• Lane No 3: Positive Control (in Vitro Methylation).

**Table 4:** Frequency Table of Methylation of *LATS2* and Smoking Status in OSCC Patients and Healthy Controls

Gene methylation status	Methylated (%)	OR (95% CI)	*P*-value
*LATS2* in Patients Samples (N= 40)	31 (77.5)	2.37 (1.26-4.45)	0.0006
*LATS2* in Control Samples (N= 7)	0	0	0.0001

**Figure 4:**
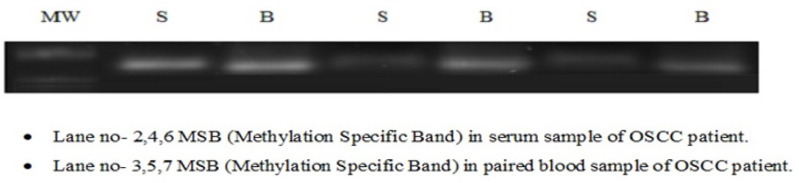
MSB (Methylation Specific Band) of LATS2 in Serum and Paired Blood Samples of OSCC Patients along with Ladder. The size of amplified product was observed to be148bp

## Results

*Isolation of DNA*

The DNA isolated from the blood and serum samples was run on the 1% agarose gel and visualized under gel documentation unit. This was carried out to check the quality of DNA (Figure 1).

*MSP of LATS2 Gene*

MSP was performed to check the promoter hypermethylation of *LATS2* gene in OSCC patients among the North Indian population.

*Status of Promoter Hypermethylation*

Hypermethylation of *LATS2* was observed in 55% of OSCC patients. 38 out of 70 samples were detected to be promoter hypermethylated through MSP (Methylation Specific PCR) in OSCC patients among north Indian population (Figure 2). The statistical analysis of promoter hypermethylation of *LATS2* gene was found to be significant (p-value= 0.0007) with an odds ratio of 1.83 (Table 2). Hypermethylation of *LATS2* gene was also observed in Serum samples although the intensity of MSB was found to be less in serum samples(Figure4).

*Correlation Between Clinicopathological Information And Methylation Status In OSCC*

The hypermethylation of promoter region of *LATS2* gene was also found to be statistically significant in tobacco chewers (Table 3) and smokers (Table 4) as the p value was found to be less than 0.05.

## Discussion

Oral squamous cell carcinoma (OSCC) is one of the most popular types of oral neoplasm, accounting for over 90 % of all mouth fatalities and 38 % of head and neck neoplasms. In fact, despite advances in the area of oral cancer detection, prevention, and multimodality approaches, the overall 5-year survival for OSCC remains to be modest at best. The common significant factor influencing OSCC survival after treatment is the grade of the Tumor at diagnosis. Hence, to promote long-term results, an immediate detection in combination with primary and secondary control strategies is critical. A methyl group is covalently joined to cytosine C5. When DNA is treated with bisulfite, unmethylated cytosines are converted to uracil, but methylated cytosines are protected. In this phenomenon, the promoter region of the gene is hypermethylated which causes global hypomethylation to the gene. When these methyl groups are segregated from the cytosine nucleotide then the expression of the sequence is altered. This is called a reversal of methylation. This cause the expression to express which is delayed due to methylation. Epigenetic changes vary from population to population as they are mainly dependent upon the environmental and dietary factors which differ greatly (Jha et al., 2017).

Previous studies have indicated the promoter methylation profiles of P14ARF, MGMT, CDH1, APC, ATM, P15INK4b, P16INK4a, FADD, FAS, ERK and RAF1 in OSCC (Kordi-Tamandani et al., 2010b; Rigi-Ladiz et al., 2011; Kordi-Tamandani et al., 2012; Saberi et al., 2014; Kordi-Tamandani et al., 2014). To the best of our knowledge, no published work is there from India on the promoter hypermethylation of the selected gene. So, the present research also focused on correlating the methylation status of *LATS2* gene with these risk factors like tobacco chewing and smoking in OSCC patients among north Indian population. In the present study, the DNA was isolated from the collected blood samples of a cancer patient (OSCC) and control samples (patients not suffering from cancer). Moreover, isolation of DNA was followed by sodium bisulfite modification by agarose bead method and E Z DNA Gold Methylation Kit (Zymo Research, US), after which MSP was performed. The statistically significant difference in methylation of *LATS2* (p-value < 0.05) between patient and control samples was observed. The methylation of *LATS2* showed the risk of developing OSCC in patients. It has been proved that the outcome novel methylation markers may be used for designing drugs that modify methylation statuses in OSCC (Mikeska and Craig, 2014.) Hence, based upon the present study *LATS2* could be used as a diagnostic biomarker in OSCC patients among north Indian population. Investigations on broader sample size are required to declare the clinical applicability of *LATS2* hypermethylation in larger groups of patients. Early estimation of *LATS2* hypermethylation might allow the identification of subgroups of patients with poor diagnosis, who might require a diverse therapeutic strategy. Therefore, the future study needs to be carried out to explore the status of this potential biomarker in the clinical control of OSCC and to estimate whether it can contribute to personalized treatment strategies.

*Conclusion and Future Perspectives*

Promoter methylation of Tumor Suppressor genes is a critical factor in carcinogenesis of OSCC. Study of DNA methylation is a beneficial procedure for the evaluation of the biological characteristics of oral cancers and may be a useful diagnostic biomarker. Hence, the present study was intended to investigate the methylation status of *LATS2* gene and to correlate the methylation status of these genes with the risk of OSCC statistically. The risk of OSCC was also calculated and was found to be significant. This confirms that *LATS2* hypermethylation is an important step in the OSCC patients among north India population and it can probably be used as a diagnostic biomarker in OSCC patients. Further the study on reversal of hypermethyation of *LATS2* gene and its reactivation needs to be carried out and can provide a good breakthrough in the study involving cancer therapy Thus research needs to be carried out on a large scale as the sample size in the present study was small to draw any complete statistical conclusion. However this study is very important as this is the first study on the methylation status of this gene in OSCC among north Indian population. 

*Abbreviations*

OSCC- Oral Squamous Cell Carcinoma; DNA- Deoxyribonucleic acid; LATS-Large Tumor Suppressor (gene); MSP-Methylation-Specific Polymerase Chain Reaction
